# Drought Stress Pre-Treatment Triggers Thermotolerance Acquisition in Durum Wheat

**DOI:** 10.3390/ijms23147988

**Published:** 2022-07-20

**Authors:** Mariarosaria De Pascali, Monica De Caroli, Alessio Aprile, Antonio Miceli, Carla Perrotta, Mariolina Gullì, Patrizia Rampino

**Affiliations:** 1 Department of Biological and Environmental Sciences and Technologies, University of Salento, Via Monteroni 165, 73100 Lecce, Italy; mariarosaria.depascali@unisalento.it (M.D.P.); monica.decaroli@unisalento.it (M.D.C.); alessio.aprile@unisalento.it (A.A.); antonio.miceli@unisalento.it (A.M.); carla.perrotta@unisalento.it (C.P.); 2 Department of Chemistry, Life Sciences and Environmental Sustainability, University of Parma, Parco Area delle Scienze 11/A, 43124 Parma, Italy; mariolina.gulli@unipr.it

**Keywords:** acquired thermotolerance, combined stress, drought priming, HSPs, *Triticum durum*

## Abstract

Durum wheat is strongly affected by climatic constraints such as high temperatures and drought, which frequently lead to yield reduction. Damages due to high temperatures are related to plant thermotolerance, a trait determined by two components: basal and acquired thermotolerance. In this study, the effect of drought and heat stress imposed singularly or sequentially was investigated in ten durum wheat cultivars (cvs) at the physiological and molecular level. The traits analyzed were cell membrane stability, relative water content, proline content, and expression level of several genes for heat shock proteins (HSPs). Our results indicate that drought priming can induce the acquisition of thermotolerance in most cultivars already classified as able to acquire thermotolerance by heat pre-treatment. Proline accumulation was correlated to cell membrane stability, meaning that the most thermotolerant cvs were able to accumulate higher levels of proline. Acquired thermotolerance is also due to the activation of *HSP* gene expression; similarly, pre-treatment with water stress was able to activate *HSPs* expression. The results reported indicate that water stress plays an important role in inducing thermotolerance, comparable to mild heat stress pre-treatment. This is the first report on the effect of drought stress on the acquisition of thermotolerance.

## 1. Introduction

Environmental changes, such as extreme temperature and reduced rainfall, have become an increasing danger for plant development and productivity. Consequently, plants growing in field conditions are increasingly exposed to abiotic stress factors. High temperatures and drought are two major environmental factors limiting crop growth and yield [1,2,3], and are predicted to become primary constraints on food production in the near future [4].

In open field conditions, high temperatures and drought are rarely present individually, and crops are often subjected to both adverse conditions, occurring sequentially or simultaneously. Plant adaptation to the different stress conditions requires activation of specific responses at the molecular, cellular and physiological levels, aimed at minimizing damages. It has been reported that plants previously exposed to a unique stress can become more resistant to subsequent stress events, through mechanisms responsible for tolerance, acquiring a kind of stress memory (priming) [5]. Moreover, when a plant is exposed to a specific abiotic stress, it can develop an increased tolerance to different abiotic stresses. This phenomenon is known as cross-stress tolerance [6]. Priming is due to transient or permanent changes at the molecular, epigenetic, biochemical and physiological levels [7]. Although the presence of common factors in the induction of cross-tolerance is well established, the elucidation of the molecular mechanisms responsible for the acquisition of cross-stress tolerance is still lacking [8,9]. It has been observed in numerous plant species that drought priming improves tolerance to a different abiotic stress, such as heat. Many data reported have indicated that the enhanced tolerance brought about by drought priming could be due to the maintenance of important pathways, including activation of antioxidant defense systems, accumulation of compatible solutes as well as photosynthesis increase [10,11,12,13]. A comprehensive understanding of the impact of heat and drought stresses is mandatory for the evaluation of the effect of climate changes on the production of important crops, such as durum wheat, in the European Mediterranean area, where crop losses due to drought and heat have tripled over the last five decades [14].

Durum wheat, *Triticum turgidum* L. var. *durum*, is a rain-fed crop widely cultivated over the Mediterranean Basin. The major climatic constraints to durum wheat yield are high temperatures and drought, frequently occurring during the crop’s growth cycle [15,16,17]. The foreseen climate changes in this region, in particular rising temperatures and decreasing rainfall [18,19], may seriously compromise durum wheat yield, and represent a serious threat to the cultivation of this typical Mediterranean crop. As reported above, the two stresses cause several biochemical and physiological changes at the cellular and whole plant levels, strongly affecting yield and quality [20].

In durum wheat, high temperatures induce damages whose level is related to the plants’ thermotolerance. Thermotolerance has two components due to different genetic bases: (i) basal thermotolerance, that is the result of evolutionary thermal adaptation to environment and (ii) acquired thermotolerance, due to the ability of genotypes to acclimate i.e., to survive lethal temperatures after exposure to mild temperature pre-treatment [21]. Genetic variability of basal and acquired thermotolerance can be screened by the evaluation of cell membrane stability (CMS). The cellular membrane damage due to stress results in increased permeability and leakage of ions, which can be readily measured by the efflux of electrolytes. Hence, the estimation of CMS by measuring cellular electrolyte leakage is finding a growing use as a screen for stress resistance. In many reports, high CMS values were found to be associated across different genetic materials with the ability to cope with stress [21,22,23,24,25].

To counteract the negative effects of heat stress at the cellular level, plants modify their metabolic pathways through mechanisms aimed at the modification of signaling and control of stress-responsive gene expression. Induction by different abiotic stress priming of accumulation of endogenous osmoprotectants to confer cross-stress tolerance has been reported in different plant species [5]. Proline is recognized as an important antioxidant and is also involved in the regulation of gene transcription to enhance stress tolerance [5,26].

Tolerance to both heat and drought stress is mediated by molecules involved in signal transduction that leads to the activation of specific gene networks, resulting from the re-programming of cell expression machinery. In particular, heat and drought tolerance is related to gene expression and accumulation of specific proteins, namely heat shock proteins (HSPs), involved in the maintenance, stabilization, and protection of membranes and proteins [27,28].

The aim of this study was to explore the effect of drought stress on thermotolerance acquisition in durum wheat. The effect of drought and heat stress imposed singularly and/or sequentially was investigated in ten different *T. durum* cultivars at the physiological and molecular levels. The data obtained help to understand the mechanisms involved in thermotolerance acquisition through pre-exposure to moderate high temperature or through water stress pre-treatment.

## 2. Results

### 2.1. Physiological Analyses

The response to the different stress treatments was analyzed in ten durum wheat cvs (namely: Ardente, Cappelli, Claudio, Colosseo, Kofa, Meridiano, Neodur, Ofanto, Simeto, Svevo) using physiological tests, allowing for the evaluation of the effects of different stress treatments on seedlings. These cvs were chosen because they exhibited different basal thermotolerance as well as dehydration tolerance in a previous study [29].

#### 2.1.1. Cell Membrane Stability (CMS) Evaluation

The ten durum wheat cvs used in this study were characterized for their heat stress tolerance based on the Cell Membrane Stability (CMS) test. The treatments utilized allowed to evaluate the constitutive thermotolerance (ThC), but also the ability to acquire thermotolerance after a pre-treatment at 34 °C for 24 h (ThA_34), after a pre-treatment of 2 h at 40 °C (ThA_40), or after drought stress pre-treatment (dehydration at room temperature for 2 h, ThA_D).

**Figure 1:**
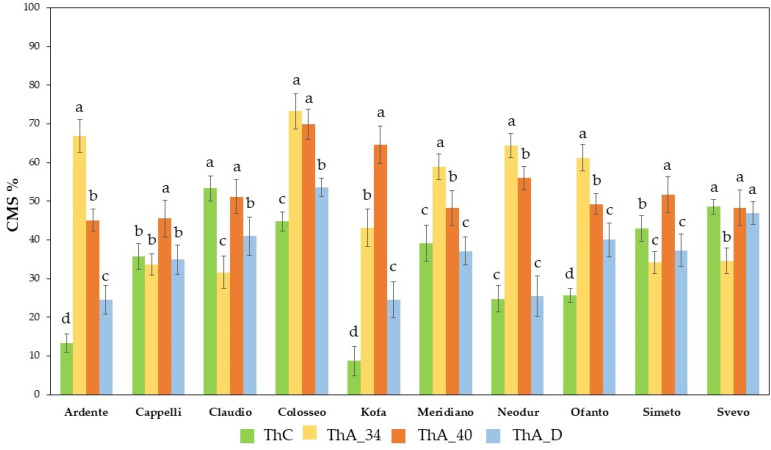
Thermotolerance of ten *T. durum* cvs evaluated using the CMS test. Individual bars represent the CMS values determined as: constitutive thermotolerance (ThC, green bar); acquired thermotolerance after a treatment at 34 °C for 24 h (ThA_34, orange bar); after heat shock at 40 °C for 2 h (ThA_40, red bar); after drought stress for 2 h (ThA_D, blue bar). The results are shown as the mean ± SD of ten independent measurements. For each cv, columns marked with different letters indicate a significant difference among treatments at *p* < 0.05 (ANOVA with post-hoc Bonferroni-Holm test).

As shown in Figure 1, the cvs differ for their ability to acquire thermotolerance. A two-way ANOVA test was performed to analyze the effect of genotype and treatment on CMS value; it revealed a statistically significant correlation between the effects of genotype and treatment (F (36, 295) = 18.9, *p* < 0.001). Simple main effects analysis showed that both genotype and treatment did have a statistically significant effect on CMS (*p* < 0.0001) (Table S1).

CMS values obtained in control conditions, corresponding to the so-called basal or constitutive thermotolerance (ThC), ranged between a minimum value of 8.74, exhibited by cv Kofa, and a maximum value of 53.28, exhibited by cv Claudio (Figure 1). After a treatment at 34 °C for 24 h (H34), thermotolerance acquisition (ThA_34) was determined, and the mean values ranged between the lowest, 31.64 exhibited by cv Claudio, and the highest, 73.21 exhibited by cv Colosseo (Figure 1). After acclimation, some cvs, i.e., Ardente, Colosseo, Kofa, Meridiano, Neodur, and Ofanto, exhibited CMS values significantly higher than in control conditions, indicating their ability to acquire thermotolerance. On the contrary, the cvs Claudio, Simeto, and Svevo exhibited CMS values significantly lower than that reported as ThC, indicating that acclimation was not able to induce thermotolerance acquisition; cv Cappelli showed similar CMS values in ThC and ThA_34. Ardente, Colosseo, Neodur, and Ofanto cvs reached a CMS above 60%; Kofa and Meridiano cvs had a CMS above 40%, while Cappelli, Claudio, Simeto, and Svevo cvs showed values below 40% and were characterized by a ThC higher than ThA_34 (similar data had been obtained for Svevo and Claudio cvs, as reported by Rampino and coworkers) [23].

When CMS was measured after pre-treatment of 2 h at 40 °C (ThA_40, Figure 1), a group of five cvs, Ardente, Cappelli, Meridiano, Svevo, and Ofanto, showed values below 50% (45.10%, 45.52%, 48.31%, 48.34%, 49.21% respectively); the other group of cvs, i.e., Claudio, Neodur, and Simeto, had CMS values between 50 and 60% (51.15%, 55.97%, 51.64% respectively); the highest values (over 60%) were observed in Kofa and Colosseo cvs (64.54% and 68.89%, respectively). Moreover, Cappelli, Kofa, and Simeto cvs reached CMS values significantly higher than in all the other treatments (Table S2). The CMS values reached by Ardente, Meridiano, Neodur, and Ofanto were significantly lower with respect to those obtained after the pre-treatment at 34 °C for 24 h (ThA_34).

The effect of drought stress pre-treatment (dehydration at room temperature for 2 h, D) on thermotolerance acquisition showed many differences among the analyzed cvs (Figure 1). In particular, Ardente, Colosseo, Kofa, and Ofanto cvs exhibited CMS values significantly higher in ThA_D than ThC, confirming their ability to also acquire thermotolerance after this kind of pre-treatment; on the contrary, the other cvs exhibited values lower or similar to that registered for ThC, indicating their inability to acquire thermotolerance. The lowest value, corresponding to 24.48, was exhibited by cv Kofa, while the highest value, attributed to cv Colosseo, was 53.57. Svevo had a unique behavior, showing similar CMS values in ThC, ThA_40, and ThA_D, although in response to acclimation at 34 °C for 24 h (ThA_34), its CMS value was significantly lower.

**Figure 2:**
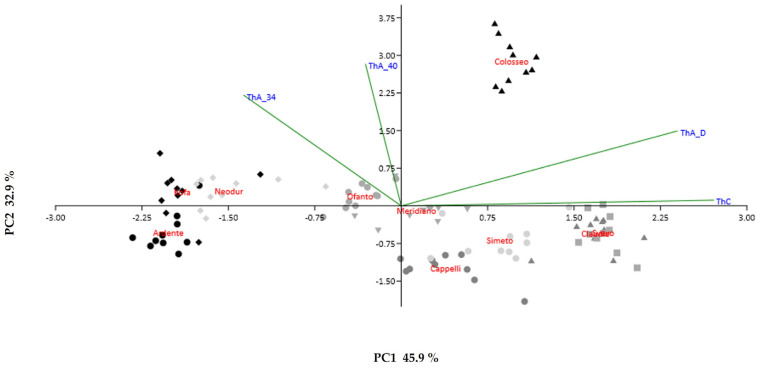
Principal component analysis (PCA) biplot of CMS measured in the ten *T. durum* cvs grown under the different stress treatments. The percentage of total variance as explained by each axis is shown, sample differentiation pattern is mostly influenced by constitutive thermotolerance (ThC) along PC1 and by acquired thermotolerance (ThA) along PC2. ThC, constitutive thermotolerance; ThA_34, acquired thermotolerance after a treatment at 34 °C for 24 h; ThA_40, acquired thermotolerance after heat shock at 40 °C for 2 h; ThA_D, acquired thermotolerance after drought stress for 2 h.

Principal component analysis (PCA) revealed patterns in plant thermotolerance across all samples (Figure 2). The scatterplot showed groups of cultivars, and the sample differentiation pattern is mostly influenced by constitutive (ThC, 45.9%) and acquired (ThA_34 32.9%) thermotolerance. Svevo and Claudio cvs are clustered together along PC1, and they are the cvs exhibiting the highest ThC. CMS values remained almost constant also in response to the other treatments (ThA_D, ThA_40). Colosseo differs from all the other cultivars and is characterised by the highest ThA_34, which is similar to ThA_40 and ThA_D.

In this set of cvs, it was interesting to observe that constitutive thermotolerance (ThC) was significantly correlated to acquired thermotolerance induced by D (ThA_D) (*p* < 0.0001), and negatively correlated to the acquired thermotolerance induced by H34 (ThA_34) (*p* = 0.0014).

#### 2.1.2. Relative Water Content (RWC) Evaluation

**Figure 3:**
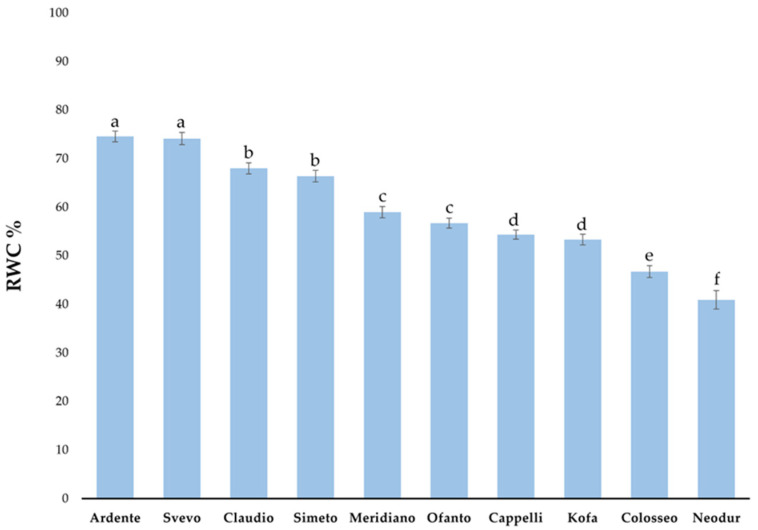
Relative water content (RWC) measured in seedlings of ten *T. durum* cvs in response to dehydration for 2 h. Different letters indicate a significant difference at *p* < 0.05 (Anova, Bonferroni-Holm’s post-hoc test).

RWC provides a measurement of the ‘water deficit’ of the leaf, and may indicate a degree of stress expressed under drought or heat stress. A Relative Water Content (RWC) test was performed on wheat seedlings subjected to dehydration for two hours (D). The results, reported in Figure 3, show that in general, RWC values declined after D stress treatment and ranged from 74.6% to 40.95%. In particular, cvs Ardente and Svevo showed high RWC values (74.6% and 74.2% respectively); the lowest values were shown by cvs Colosseo and Neodur (46.8% and 40.9% respectively).

#### 2.1.3. Free Proline Content Evaluation

**Figure 4:**
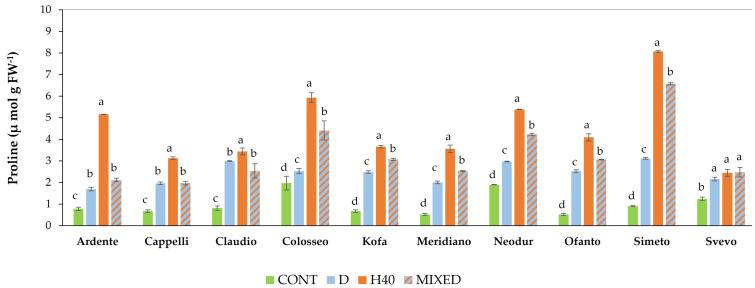
Free proline content measured in ten *T. durum* cvs in control conditions (CONT, green bar) and in response to drought stress (D, blue bar), to heat shock (H40, red bar), to mixed stress (MIXED, blue-red bar). The results are shown as the mean and standard deviation of the three replicates, expressed in μ mol g FW^−1^. For each cv, columns marked with different letters indicate significant difference among treatments at *p* < 0.05 (ANOVA with post-hoc Bonferroni-Holm test).

Free proline content was evaluated in all wheat cvs in response to different stress treatments, namely D, H40 (40 °C for 2 h) and MIXED (2 h of drought followed by 2 h at 40 °C) (Figure 4). A two-way ANOVA test was performed to analyze the effect of genotype and treatment on proline content, revealing a statistically significant interaction between the effects of genotype and treatment (F (27, 80) = 116, *p* < 0.001). Simple main effects analysis showed that both genotype and treatment did have a statistically significant effect on free proline content (*p* < 0.0001) (Table S3).

The amount of free proline in the control samples was minimum in all cvs, but Colosseo and Neodur showed a significantly higher amount than the other cvs (*p* < 0.05) (Table S4). In all cvs, the highest amount of proline was observed after the treatment H40; Simeto showed the highest amount of proline (8.08 μ mol g FW^−1^) and Svevo the lowest (2.45 μ mol g FW^−1^). In all cvs, the amount of proline measured at H40 was significantly higher compared to the other treatments, with the exception of Svevo, in which the amount of proline measured in response to all stress treatments was similar, although still significantly higher compared to the control condition.

In response to D, Simeto and Claudio showed the highest amount of proline (3.12 and 3 μ mol g FW^−1^ respectively), while Ardente had the lowest one (1.69 μ mol g FW^−1^). In response to a MIXED stress, Simeto showed the highest amount of proline (6.57 μ mol g FW^−1^) and Cappelli the lowest one (1.97 μ mol g FW^−1^). The amount of proline measured in response to MIXED was intermediate between H40 and D treatments in cvs Colosseo, Kofa, Meridiano, Neodur, Ofanto, and Simeto. In cvs Ardente, Cappelli, Claudio, and Svevo, D and MIXED stresses determined a similar increase of free proline, significantly higher as compared to control condition (*p* < 0.05) (Figure 4). Interestingly, a positive correlation was observed between proline accumulation and CMS, meaning that the most thermotolerant cvs were able to accumulate high levels of proline, while a negative correlation was observed for RWC (Table 1).

### 2.2. Molecular Analysis

**Figure 5:**
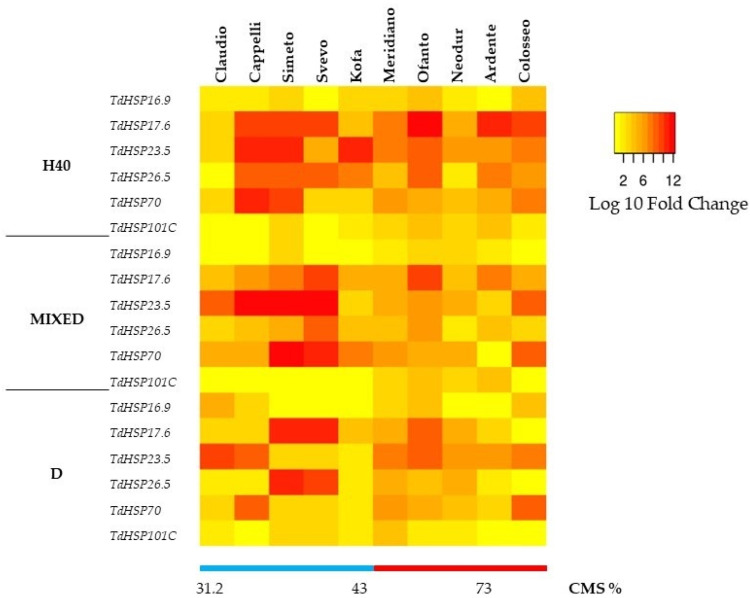
Heat-map showing the relative expression of *TdHSP16.9, TdHSP17.6, TdHSP23.5, TdHSP26.5, TdHSP70* and *TdHSP101C* measured in wheat seedlings of ten different cvs in response to heat stress (2 h at 40 °C = H40), MIXED stress (drought stress 2 h followed by heat stress 2 h at 40 °C), and to drought stress (2 h = D). Data are expressed as log10 Fold-change (FC). The order of cvs is related to their acquired thermotolerance (ThA_34) evaluated by CMS test.

The effect of stress at the cellular level was analyzed by quantitative PCR to determine variations in *HSPs* transcript level. Real time PCR analysis was performed on RNAs extracted from control seedlings and from seedlings subjected to heat stress (2 h at 40 °C, H40), mixed stress (drought stress 2 h followed by heat stress 2 h at 40 °C, MIXED), and to drought stress (dehydration for 2 h, D). The genes analyzed were: *TdHSP16.9* (class I cytosolic), *TdHSP17.6* (class II cytosolic), *TdHSP23.5* (mitochondrial), *TdHSP26.5* (plastidial), *TdHSP70* (cytosolic), and *TdHSP101C* (cytosolic), and the results obtained are reported in Figure 5.

The expression pattern was different among the genes analyzed as well as among various cvs. Each *HSP* gene was expressed at a different level in relation to stress severity and genotype. In general, all the stress treatments induced *HSP* transcript accumulation. The treatment inducing the highest accumulation level was heat stress (H40) for almost all the cvs. When seedlings were subjected to the MIXED stress, accumulation of transcripts was lower than after H40, while, in response to D, the induction was lower as compared to H40 or to MIXED stresses.

*TdHSP17.6 TdHSP23.5*, *TdHSP26.5* and *TdHSP70* showed high induction levels in response to all stresses, with decreasing values from H40, to MIXED and to D; overall these genes were the most stress-responsive in the cvs characterized by low level of acquired thermotolerance (CMS below 50%), which are Cappelli, Claudio, Kofa, Simeto, and Svevo. *TdHSP16.9* was characterised by a low level of induction in all cvs as well as in all stress conditions.

*TdHSP101* was induced in response toH40 and to the MIXED stress, particularly in cvs with a medium/high level of thermotolerance (CMS > 50%) as Ardente, Colosseo, Meridiano, Neodur, and Ofanto, while in response to D, a limited induction was observed in all cvs (Log10 FC in the range 0.3–4.1).

## 3. Discussion

Natural environments, especially when characterized by low water and high temperature, induce stress in plants causing reduced growth, development, and productivity. In durum wheat, which grows in the Mediterranean area, the ability to cope with drought and high temperature stresses is particularly important for minimizing the effect of global warming on productivity. The wide variability to cope with these stresses [17,30] is based on differences in the genetic background that influence the ability to acquire tolerance; also pre-exposure to the same or to a different abiotic stress is able to induce tolerance.

In this work, we characterized 10 durum wheat cultivars for their basal and acquired thermotolerance using a CMS test. The results of this analysis indicate variability in the genotypes analyzed for both basal and acquired thermotolerance, and allowed for classification according to thermotolerance acquisition capability. The data reported indicate that thermal pre-treatments (H34 or H40) induce thermotolerance at different levels in the genotypes that are able to acquire thermotolerance.

It is known that plants pre-exposed to the same kind of stress or to another abiotic stress can acquire tolerance to subsequent stresses through the so-called priming mechanism. It was also reported that drought priming improves tolerance to various stresses (heat, chilling, drought) in different plant species, although the mechanisms by which drought priming enhances thermotolerance are not yet understood [31]. In order to explore whether a pre-treatment with drought stress is also able to induce thermotolerance in durum wheat, CMS was evaluated after drought priming (D). Our data indicate that drought priming induces the acquisition of thermotolerance, although at lower level, in most cvs already classified as able to acquire thermotolerance by heat pre-treatment. Genotypes classified as unable to acquire thermotolerance by heat pre-treatment also confirmed their inability to acquire thermotolerance after drought priming.

Proline accumulation in response to heat stress is reported as a species-specific characteristic, with some species showing increased accumulation and others exhibiting decreased accumulation. As an example, in cotton proline, accumulation following heat priming was able to increase salt and drought tolerance [32]; in barley, proline accumulation after cold priming was associated with freezing tolerance. In germinating wheat seeds exposed to high temperatures, a reduced proline biosynthesis was observed [33]. In soybean, modification of proline levels by genetic modification highlighted the protective role of proline against simultaneous drought and heat stress [34].

Evaluation of proline accumulation after different stress treatments in the durum wheat cvs utilized in this study indicates that this osmolyte accumulates after heat stress at higher levels than after drought-primed heat stress. In addition, high variability was observed among the cultivars; interestingly, a positive correlation was observed between proline accumulation and CMS, meaning that the most thermotolerant cvs were able to accumulate a high level of proline, while a negative correlation was observed for RWC, which is expected, since proline biosynthesis is activated during dehydration [35].

Another aspect of cell response to stress is the modulation of specific genes (*HSPs*) already known to be involved in plant molecular response to abiotic stress. In general, HSPs are considered chaperones involved in the regulation of cellular homeostasis under stress conditions [36]. Moreover, the level of *HSP* expression has been reported to correlate with abiotic stress tolerance [28,30,37].

Many studies have suggested the importance of HSPs in the crosstalk among cellular response networks activated by different abiotic stresses [37,38]. HSPs are also known to play a pivotal role in cross-stress tolerance, as demonstrated by transgenic plant studies indicating that *HSP* over-expression implies the activation of other stress response genes, high antioxidant activity, and high osmolyte levels [5,39,40].

In this work, we analysed the expression of *HSP* family members already known to be involved in thermotolerance acquisition mechanisms. The target genes considered were: (i) components of plant small *HSP* family (cytosolic, mitochondrial, and plastidial) coding for proteins acting as chaperones able to prevent cellular protein aggregation and, at the same time, to help cell proteins correct refolding [41]; (ii) a member of the *HSP70* family exhibiting a role in heat tolerance in various plant species [25]; (iii) a *HSP* gene coding for the high molecular weight HSP, HSP101C, a key stress protein involved in the acquisition of stress tolerance under different stress conditions [42,43]. All the *HSP* genes analysed were up-regulated in response to the different stress conditions, at different levels, depending on the genotype.

Several studies have addressed plant response to multiple stresses occurring simultaneously, and are reported in a recent review [44], while specific studies on the effect of drought on the acquisition of thermotolerance are still lacking.

Our results indicate that, in durum wheat, the same way pre-treatment at moderate temperatures induces thermotolerance through the activation of *HSP* gene expression, pre-treatment with water stress is able to activate *HSP* expression, thus inducing thermotolerance. This observation could imply that the *HSP* expression, induced by drought stress pre-treatment, may have a role in protecting the plants during the subsequent heat stress.

Although further investigations are needed to identify specific factors modulating the molecular basis of thermotolerance, the findings reported here contribute to the addition of new knowledge about this phenomenon.

## 4. Materials and Methods

### 4.1. Plant Materials and Stress Treatments

Seeds of 10 different wheat (*Triticum durum* L., var. *durum*) cultivars (cvs), namely: Ardente, Cappelli, Claudio, Colosseo, Kofa, Meridiano, Neodur, Ofanto, Simeto, and Svevo, were used. Seedlings were grown in PERLIGRAN (Deutsche Perlite, Dortmund, Germany) at 25 °C under a constant light/dark regime with 16 h light and 8 h darkness, and watered daily with tap water. Ten-day-old seedlings exhibiting comparable leaf size were selected and used for physiological and molecular analyses after different stress treatments. Well-watered 10-day-old seedlings were harvested and used as control; for drought stress (D), 10-day-old seedlings were placed on dry filter paper for 2 h at room temperature (23–25 °C); for heat stress (HS), well-watered seedlings were subjected either to 34 °C for 24 h (H34) or to 40 °C for 2 h (H40); for drought and heat sequential stress (MIXED), seedlings were placed on dry filter paper for 2 h at room temperature and then for 2 h at 40 °C. At the end of each stress treatment, seedlings were subjected to physiological tests or frozen in liquid nitrogen for subsequent molecular analysis.

### 4.2. Evaluation of Cell Membrane Stability

Cell Membrane Stability (CMS) was evaluated as previously reported (Rampino et al., 2020); 3.5 cm long leaf segments were rinsed in distilled water, placed in a closed tube with 2 mL of distilled water, and incubated at 50 °C for 1 h (T1), while controls were kept at 10 °C (C1). 8 mL of distilled water were then added to each tube and the tubes were incubated at 10 °C for 24 h. When samples reached room temperature, conductivity of the solution was measured using a Crison GLP 31 conductivity meter (Crison Instruments, Barcelona, Spain). Samples were autoclaved at 100 °C/0.10 MPa for 15 min (T2, C2) and the conductivity was measured again. Ten replicates for each cultivar (cv) were analyzed, and CMS was calculated as follows:

CMS (%) = [1 − (T1/T2)/1 − (C1/C2)] × 100

### 4.3. Measurement of RWC

Measurement of leaf Relative Water Content (RWC) was performed on control and stressed seedlings as previously reported [29]. Fully expanded leaves from control and stressed plants were excised, and fresh weight (FW) was recorded; excised leaves were soaked for 4 h in distilled water at room temperature, and the turgid weight (TW) was recorded. Drying was obtained by exposure at 80 °C for 24 h, and dry weight (DW) was recorded. RWC was calculated following the formula:

RWC (%) = [(FW − DW)/(TW − DW)] × 100

Ten replicates for each cv were analyzed.

### 4.4. Free proline Determination

Frozen plant material (0.5 g) from control and stressed seedlings was homogenized after adding 10 mL of 3% sulfosalicylic acid; the homogenate was filtered through Whatman filter paper. Free proline amount was measured according to Bates and coworkers [45] and reported as μmol g FW^−1^. In a test tube, 2 mL of filtrate were added to 2 mL of acid-ninhydrin and 2 mL of glacial acetic the reaction was performed at 100 °C for 1 h and stopped in an ice bath. To the reaction mixture 4 mL toluene were added and vigorously mixed for 15–20 s. The organic phase was aspirated, warmed to room temperature and the absorbance was determined at 520 nm. The proline concentration was derived from a standard curve obtained using reference proline solutions.

### 4.5. RNA Extraction, cDNA Synthesis and Quantitative RT-PCR

Total RNA was isolated using the “SV Total RNA Isolation System” (Promega, Madison, WI, USA) according to supplier’s instructions. Concentration and purity of extracted RNA were spectrophotometrically estimated and were confirmed by agarose gel electrophoresis. Synthesis of cDNA was performed by SuperScript III Reverse Transcriptase (Invitrogen, Carlsbad, MA, CA) following supplier’s instructions, using oligo(dT)_15_ primer; cDNA was quantified by Qubit fluorometer™ (Invitrogen) and stored at −20 °C.

Quantitative RT-PCR (qRT-PCR) was performed in 96-well plates using the SYBR Green fluorescent detection method in a Real-Time PCR thermal cycler (QuantStudio™ 3 Real-Time PCR System, Applied Biosystems). Primers were designed on the basis of *Triticum* sequences previously reported [23,25], selected using ‘Primer3Plus’ software (http://www.bioinformatics.nl/cgi-bin/primer3plus/primer3plus.cgi, accessed on 31 January 2022), and are reported in Table S5 The PCR program was as follows: 2 min at 50 °C and 10 min at 95 °C, followed by 45 cycles of 95 °C for 15 s and 60 °C for 1 min. In this work, we used α-Tubulin as reference gene, each qRT-PCR assay consisted of three independent biological and three technical replicates. Melting curve analysis was performed to evaluate the presence of non-specific PCR products. Comparative Ct method calculation steps to calculate the fold changes (FC) consist of the following three steps: (1) normalization to endogenous control by comparison of target gene and endogenous control ∆Ct  =  Ct target gene-Ct endogenous gene; (2) normalization to calibrator sample ∆Ct sample-∆Ct calibrator  =  ∆∆Ct; (3) using the formula 2^−∆∆Ct^ [46].

### 4.6. Statistical Analysis

Data are presented as means ± standard deviations (SD), which were calculated using Microsoft Office Excel 2013 software (Microsoft Corporation). The statistical significance of the data was assessed by two-way ANOVA for testing the significance between cultivars and treatments; one-way ANOVA with Bonferroni-Holm’s test was utilized to compare the response of each cv in the different stress treatments. (Daniel’s XL Toolbox, version 7.3.4, Daniel Kraus, Boston) (http://www.xltoolbox.net, accessed on 31 March 2022). *p* < 0.05 was considered to indicate statistical significance throughout the study.

Principal Components Analysis (PCA) and correlation analysis were performed using the software Past v. 3.14 [47].

Heatmap was generated using the freely available web server Heatmapper [48].

## 5. Conclusions

This paper reports the analysis performed on ten durum wheat cvs to characterize their ability to acquire thermotolerance and to respond to drought and heat stress imposed singularly and/or sequentially. The reported results indicate that water stress plays an important role in inducing thermotolerance, comparable to mild heat stress pre-treatment. To our knowledge, this is the first report on the effect of drought stress on the acquisition of thermotolerance, and, although further studies are needed to highlight molecular mechanisms underlying their response, the results obtained can be considered a starting point for future studies on the influence of drought stress on thermotolerance acquisition in durum wheat.

## Figures and Tables

**Table 1:** Pearson’s correlation coefficients between proline, CMS and RWC measured in the ten *T. durum* cvs. Bolded values correspond to *p*-values.

	Proline	CMS	RWC
Proline		**0.010961**	**0.0076678**
CMS	0.51416		**0.12543**
RWC	−0.53064	−0.3739	

## Data Availability

The original contributions presented in this study are included in the article.

## Supplementary Materials

The following supporting information can be downloaded at: https://www.mdpi.com/article/10.3390/ijms23147988/s1.
