# Supplementary material for: Drought Stress Pre-Treatment Triggers Thermotolerance Acquisition in Durum Wheat
Source: Int J Mol Sci. 2022 Jul 20;23(14):7988. doi: 10.3390/ijms23147988 (PMC9323298; doi:10.3390/ijms23147988)
Supplement: Supplementary file 1 [file ijms-23-07988-s001.zip › ijms-1795414-supplementary.pdf]

Supplementary Materials

Table S1. Two-way ANOVA REPORT for CMS measured in ten wheat cultivars in response to different treatments.

Source of variation	Sum of square	df	Mean Square	F	p-value	
Cultivar	12325.7	9	1369.5	29.7	1.3E-36	***
Treatment	19922.8	4	4980.7	108.0	1.49E-56	***
Interaction	31349.9	36	870.8	18.9	3.56E-57	***
Res. error	13605.9	295	46.1			
Total	77095.7	344	224.1			

Table S2 Thermotolerance of ten *T. durum* cvs evaluated using the CMS test. Mean values represent the CMS (%) values determined as: constitutive thermotolerance (ThC); acquired thermotolerance after a treatment at 34°C for 24 hours (ThA_34); after heat shock at 40°C for 2 hours (ThA_40); after drought stress for 2 hours (ThA_D). The results are shown as the mean ± SD of ten independent measurements. For each treatment, values marked with different letters indicate a significant difference among cultivars at p< 0.05 (ANOVA with post-hoc Bonferroni-Holm test).

	ThC		ThA_34		ThA_40		ThA_D	
	**Mean**	**SD**	**Mean**	**SD**	**Mean**	**SD**	**Mean**	**SD**
Ardente	13.38 ±	2.4 e	66.83 ±	4.2 ab	45.10 ±	2.9 c	24.51 ±	3.7 c
Cappelli	35.76 ±	3.4 c	33.63 ±	2.7 e	45.52 ±	4.8 c	34.93 ±	3.8 b
Claudio	53.29 ±	3.3 a	31.64 ±	4.2 e	51.15 ±	4.4 bc	40.96 ±	4.9 b
Colosseo	44.81 ±	2.5 b	73.21 ±	4.6 a	69.89 ±	3.9 a	53.57 ±	2.3 a
Kofa	8.74 ±	3.8 e	43.10 ±	4.8 d	64.54 ±	4.8 a	24.48 ±	4.7 c
Meridiano	39.13 ±	4.7 bc	58.90 ±	3.4 c	48.31 ±	4.4 c	37.13 ±	3.6 b
Neodur	24.79 ±	3.4 d	64.33 ±	3.2 bc	55.97 ±	3.1 b	25.39 ±	5.2 c
Ofanto	25.63 ±	1.8 d	61.23 ±	3.5 bc	49.21 ±	2.7 bc	40.02 ±	4.4 b
Simeto	42.92 ±	3.3 b	34.12 ±	2.8 e	51.64 ±	4.7 bc	37.28 ±	4.2 b
Svevo	48.59 ±	1.9 b	34.50 ±	3.3 e	48.34 ±	4.6 c	46.98 ±	2.9 b

Table S3. Two-way ANOVA REPORT for Proline measured in ten wheat cultivars in response to different treatments.

Source of variation	Sum of square	df	Mean Square	F	p-value	
Cultivar	85.2	9	9.5	514.2	6.45E-67	***
Treatment	194.1	3	64.7	3513.3	8.61E-85	***
Interaction	57.7	27	2.1	116.0	2.17E-53	***
Res. error	1.5	80	0			
Total	338.5	119	2.8			

Table S4. Free proline content measured in ten *T. durum* cvs in control conditions (CONT) and in response to drought stress (D), to heat shock (H40), to mixed stress (MIXED). The results are shown as the mean and standard deviation of the three replicates, expressed in μ mol g FW^{-1}. In each column, values marked with different letters indicate significant difference among cultivars at $p < 0.05$ (ANOVA with post-hoc Bonferroni-Holm test).

	Proline content (μ mol g FW^{-1})							
	CONT		D		H40		MIXED	
	Mean	SD	Mean	SD	Mean	SD	Mean	SD
**Ardente**	0.78 ±	0.08 b	1.69 ±	0.08 cd	5.16 ±	0.01 b	2.12 ±	0.08 d
**Cappelli**	0.67 ±	0.06 b	1.96 ±	0.06 cd	3.13 ±	0.06 d	1.97 ±	0.08 d
**Claudio**	0.81 ±	0.10 b	3.00 ±	0.02 a	3.44 ±	0.16 cd	2.54 ±	0.33 cd
**Colosseo**	1.97 ±	0.31 a	2.53 ±	0.12 c	5.93 ±	0.23 b	4.41 ±	0.45 b
**Kofa**	0.67 ±	0.06 b	2.49 ±	0.06 c	3.66 ±	0.06 c	3.07 ±	0.05 c
**Meridiano**	0.52 ±	0.05 b	2.00 ±	0.06 d	3.56 ±	0.17 cd	2.54 ±	0.02 d
**Neodur**	1.90 ±	0.01 a	2.98 ±	0.02 b	5.39 ±	0.01 b	4.23 ±	0.05 b
**Ofanto**	0.52 ±	0.05 b	2.53 ±	0.06 c	4.09 ±	0.17 c	3.06 ±	0.02 bc
**Simeto**	0.90 ±	0.03 b	3.12 ±	0.04 a	8.08 ±	0.05 a	6.57 ±	0.05 a
**Svevo**	1.24 ±	0.08 b	2.16 ±	0.08 c	2.45 ±	0.17 d	2.48 ±	0.22 d

Table S5. Primers set used for qRT-PCR. Target genes and their relative GenBank IDs are reported.

**Primer Sequence (5′- 3')**	**Target Gene (GeneBank ID)**
For-CAATGCCGGATGGACTG<br>Rev-GTGAAGGCCGGGCTGGA	*TdHSP16.9 (*AM709754.1)
For-CTCGGGTCCGGCGACA<br>Rev-CTCCGCCGTGTGCCG	*TdHSP17.6* (AJ971359.1)
For-AAGTACAACCGCCGCA<br>Rev-GAAGACGTCCTTGCGCT	*TdHSP23.5* (AM709764)
For-AGCACAAGAAGGAGGCC<br>Rev-TCACTGGACCTGCACGT	*TdHSP26.5* (AJ971373.1)
For-GCTGATTGGCAGGAGGTTCT<br>Rev-TTGTGCTTGCGCTTGAACTC	*TaHSP70* (AF005993.1)
For-CGAGAACTCCACGGTGTACATC<br>Rev-TGCTTGTCGACGCCATAGG	*TdHSP101C*(AJ970536.2)
For-AGCCAGTTCCACTCCAACA<br>Rev-GAGGATGCTGCCAACAACTTC	*TaTub* (U76558)
